# Supplementary material for: MicroRNA Expression in Clear Cell Renal Cell Carcinoma Cell Lines and Tumor Biopsies: Potential Therapeutic Targets
Source: Int J Mol Sci. 2022 May 17;23(10):5604. doi: 10.3390/ijms23105604 (PMC9147802; doi:10.3390/ijms23105604)
Supplement: Supplementary file 1 [file ijms-23-05604-s001.zip › ijms-1734714-supplementary.pdf]

RNA Precursor			Ref	Target1		Target2		Target3		Target4		Target 5	
			U6	miR17	Fold Exp	miR19a	Fold Exp	miR34a	Fold Exp	miR155	Fold Exp	miR210	Fold Exp
GU1411	STN	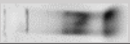	23.21	36.64	1	31.3	1	27.91	1	32.76	1	33.17	1
	STP	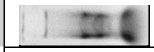	24.61	36.82	1.987902	32.03	1.601288	26.41	3.848532	29.48	7.749479	31.66	3.863695
GU1418	STN	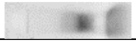	21.49	30.29	1	29.26	1	25.91	1	30.47	1	31.1	1
	STP	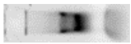	26.27	41.59	0.15632	34.14	1.951561	30.04	2.620972	33.11	4.708857	33.45	5.277642
GU1436	STN	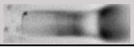	19.14	26.64	1	27.95	1	19.28	1	23.63	1	24.63	1
	STP	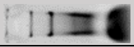	18.24	26.62	0.619197	28.17	0.563434	17.63	1.175413	21.1	1.66139	20.5	3.116805
GU1468	STN	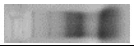	18.58	26.98	1	28.51	1	19.93	1	23.61	1	25.12	1
	STP	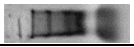	18.84	34.83	0.052547	29.66	0.732378	25.82	0.113571	29.28	0.123833	29.18	0.233229
GU1508	STN	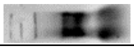	24.26	35.66	1	30.95	1	25.03	1	28.52	1	29.43	1
	STP	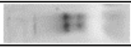	21.99	32.7	0.93718	34.73	0.066192	17.59	5.456193	19.36	10.73052	20.46	9.958037
GU1541	STN	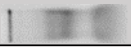	18.31	26.98	1	27.05	1	21.9	1	24.81	1	24.8	1
	STP	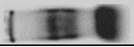	18.94	28.11	0.901853	28.14	0.916151	20.1	2.854325	24.37	1.672056	22.19	3.924914

Table S1. This table represents the number of PCR cycles for each miRs for the six patients included in the manuscript with the RNA pictures for the ribosomal RNA in the normal adjacent and the tumor tissues.
